# Differential proteomic analysis of plasma-derived exosomes as diagnostic biomarkers for chronic HBV-related liver disease

**DOI:** 10.1038/s41598-022-13272-4

**Published:** 2022-08-24

**Authors:** Bo Ye, Yifei Shen, Hui Chen, Sha Lin, Weilin Mao, Yuejiao Dong, Xuefen Li

**Affiliations:** Department of Laboratory Medicine, Key Laboratory of Clinical In Vitro Diagnostic Techniques of Zhejiang Province, The First Affiliated Hospital, College of Medicine, Zhejiang University School of Medicine, No. 79 Qingchun Road, Hangzhou, 310003 People’s Republic of China

**Keywords:** Hepatitis B, Liver cancer, Proteomic analysis, Liver cirrhosis, Risk factors

## Abstract

Hepatitis B virus (HBV) infection is still a major public health problem worldwide. We aimed to identify new, non-invasive biomarkers for the early diagnosis of chronic HBV-related diseases, reveal alterations in the progression of chronic hepatitis B (CHB), liver cirrhosis (LC), and hepatocellular carcinoma (HCC). Here, exosomes were isolated and characterized through size exclusion chromatography and nanoparticle tracking analysis. Profiles of differentially expressed proteins (DEPs) were analyzed through liquid chromatography-tandem mass spectrometry (LC–MS/MS), Gene Ontology, and Kyoto Encyclopedia of Genes and Genomes analyses. Results showed that the DEPs, including CO9, LBP, SVEP1, and VWF levels in extracellular vesicles (EVs) were significantly higher in CHB than in healthy controls (HCs). VWF expression levels in EVs were significantly lower in CHB than in those with LC. KV311 expression levels in EVs were significantly higher, whereas LBP levels were significantly lower in patients with CHB than in those with HCC. All biomarkers seemed to exhibit a high diagnostic capacity for HBV-related liver disease. Patients with HBV-induced chronic liver disease exhibit characteristic protein profiles in their EVs. Thus, serum exosomes may be used as novel, liquid biopsy biomarkers to provide useful clinical information for the diagnosis of HBV-related liver diseases at different stages.

## Introduction

Hepatitis B virus (HBV) is an epidemic worldwide. Following an infection with HBV, patients who have not cleared the virus in 6 months are diagnosed with a chronic HBV infection^1^. Chronic hepatitis B (CHB) is still a global public health problem and the overall prognosis of patients with chronic hepatitis is directly related to the severity of the disease. The World Health Organization (WHO) estimated that 257 million people were infected with chronic hepatitis in 2015, and approximately 887,000 people died from HBV infection-related diseases, with 52% and 38% of the deaths related to liver cirrhosis (LC) and hepatocellular carcinoma (HCC), respectively^2,3^. The outcome of a chronic HBV infection is variable; in case the inflammation is not suitably resolved, pathological fibrosis may develop, ultimately leading to cirrhosis and an increased risk of HCC. LC is considered to be the end stage of chronic hepatopathies which may lead to HCC^1,4,5^.

The lack of highly sensitive and specific diagnostic biomarkers is a major contributor to the poor prognosis of patients with LC or HCC^1,6^. At present, CHB, LC, and HCC are diagnosed using hematology, radiology, and histology, combined with the diagnosis of clinical symptoms. The measurement of non-specific serum biomarkers, such as alanine aminotransferase, which is used for the clinical evaluation of liver tissue, and alpha-fetoprotein (AFP), which is used for the diagnosis of HCC, is widely used for the auxiliary diagnosis of diseases. However, they are not sensitive biological indicators of liver damage. Some patients with chronic HBV infection and liver tissue damage have normal alanine aminotransferase levels^7^. Moreover, approximately one-half of patients with HCC are AFP-negative. Radiological analysis also has some limitations, because it cannot accurately determine the malignancy of the tumor mass, especially in the early stages. In addition, liver biopsy, which is the gold standard, is the only option to determine the presence of inflammation in the liver tissue. However, liver biopsy is invasive, involving the risk of serious complications, and cannot be used to evaluate dynamic changes^8^, thereby limiting its clinical application. Therefore, the identification of accurate, non-invasive biomarkers for the early diagnosis and differential detection of CHB, LC, and HCC is an urgent necessity.

Exosomes are important for the pathophysiological process of liver disease^9^. Exosomes, derived from endosomes, are membrane-bound vesicles, 40–100 nm in diameter. They participate in cell–cell communication, especially non-contact, remote regulation through proteins, mRNAs, microRNAs, and other substances contained in the vesicles^10^. Increasing evidence indicates the potential use of exosomes for the early detection of diseases^11–14^. In liver diseases, various processes, such as activation of the hepatic innate immune system, activation of hepatic stellate cells, hepatocyte apoptosis, organelle dysfunction, and systemic inflammation, may lead to the release of extracellular vesicles (EVs) ^15^. However, systematic screening of plasma exosomes for identifying the progression of chronic HBV infection has not been reported.

The aim of this study was to identify non-invasive biomarkers for the early diagnosis and differential detection of CHB, LC, and HCC, a challenging and limited area in chronic liver disease.

## Results

### Characterization of plasma-derived exosomes from patients with CHB, LC, and HCC and healthy controls (HCs)

**Figure 1:**
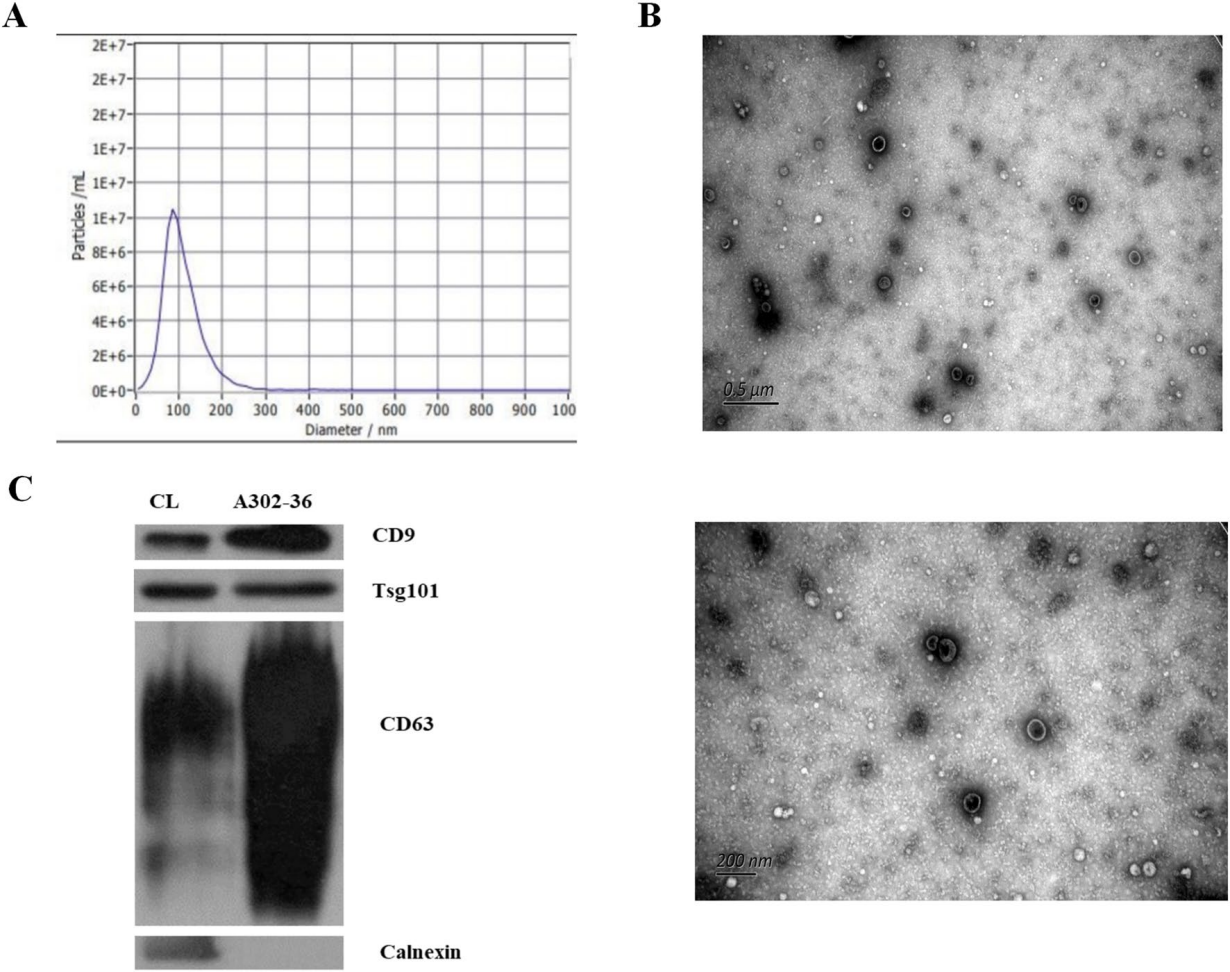
Characterization of plasma-derived exosomes from patients with chronic hepatitis B virus infection (CHB), liver cirrhosis (LC), and hepatocellular carcinoma (HCC) and healthy controls (HCs). The size and morphological distribution of exosomes are shown, and were obtained using nanoparticle tracking analysis (NTA) (**A**) and transmission electron microscopy (TEM) (**B**). The samples were positive for the exosome markers CD9, TSG101, and CD63 and negative for calnexin (**C**).

Isolated, plasma-derived exosomes were characterized using nanoparticle tracking analysis (NTA), transmission electron microscopy (TEM), and western blot (WB) analysis. The size and morphological distribution of the exosomes were evaluated using NTA and TEM. NTA results revealed that the average particle size of the exosomes in the isolated fractions was 106.9 nm (Fig. 1a). In TEM images, cup-shaped vesicles were observed (Fig. 1b). Additionally, through WB analysis, the samples were found to be positive for the exosome markers, CD9, TSG101, and CD63, and negative for calnexin (Fig. 1c).

### Profiles of differentially expressed proteins between plasma-derived exosomes from patients with CHB, LC, and HCC and HCs

**Figure 2:**
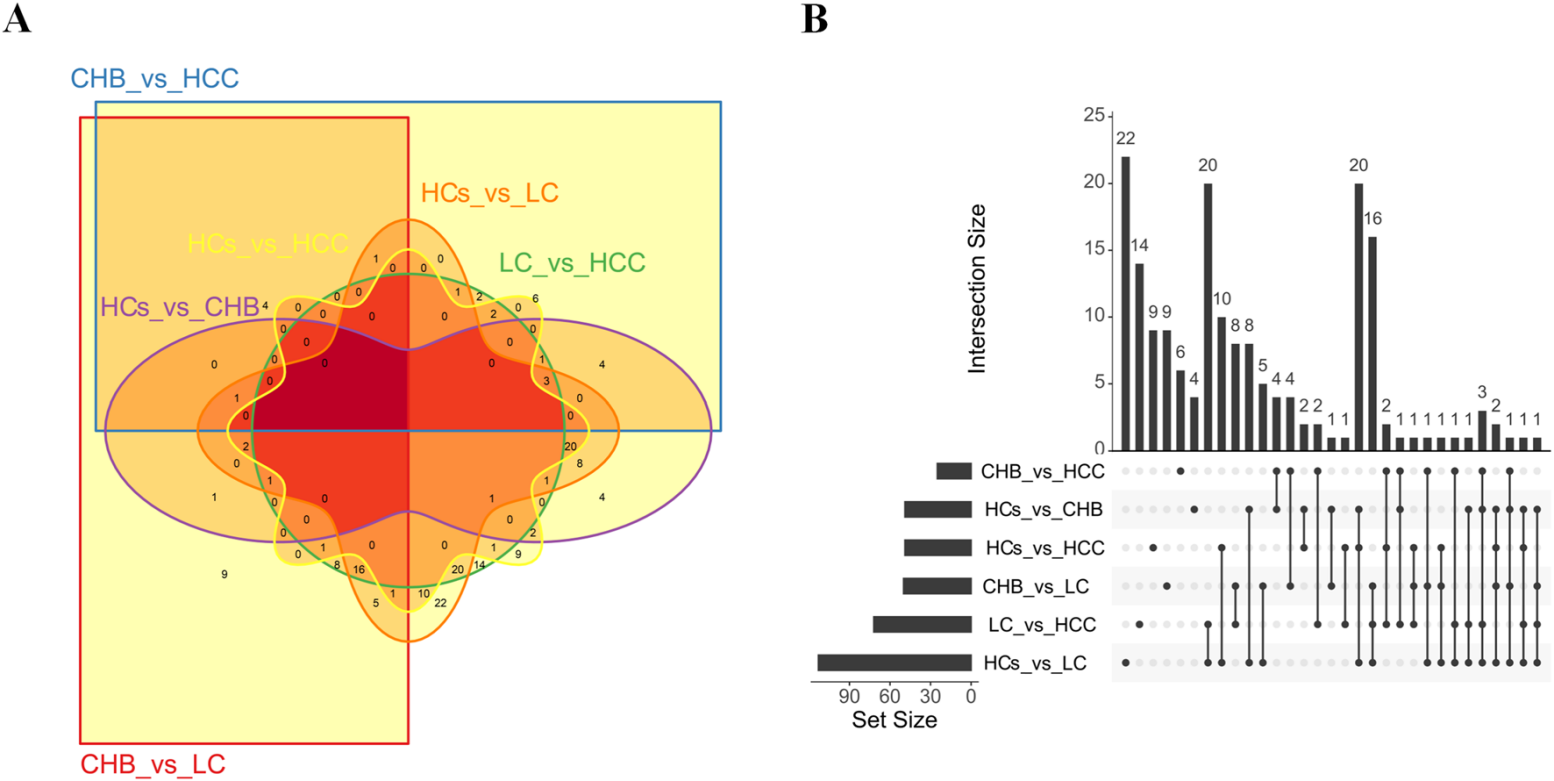
A Venn diagram showing pairwise comparison of differentially expressed proteins between patients with chronic HBV infection (CHB), liver cirrhosis (LC), and hepatocellular carcinoma (HCC) and healthy controls (HCs). (**A**) The Venn diagram was drawn by the R package Vennerable. For convenience of description, CHB, LC, HCC, and HCs groups are denoted by CHB, LC, HCC, and HCs, respectively. (**B**) An UpSet diagram showing pairwise comparison of differentially expressed proteins between CHB, LC, HCC, and HCs. The UpSet diagram was drawn by the R package UpSetR. The figure shows the number of common or unique proteins in different combinations. The lower left corner shows groups with differential expression and the number of different proteins that were screened among each group. The dots on the right show proteins common or specific to the corresponding group. The histogram shows the number of specific or common proteins.

In 28 samples, the number of peptides obtained by enzymatic hydrolysis of exosomal proteins extracted from each case ranged from 105 to 693. The number of differentially expressed proteins between pairs of groups was calculated. The highest and lowest numbers of differentially expressed proteins were 113 and 25 for HCs versus LC and CHB versus HCC, respectively. CHB, LC, HCC, and HCs were compared pairwise within six groups; the six groups of differentially expressed proteins were then analyzed using Venn diagrams and UpSet diagrams (Fig. 2a,b).

**Figure 3:**
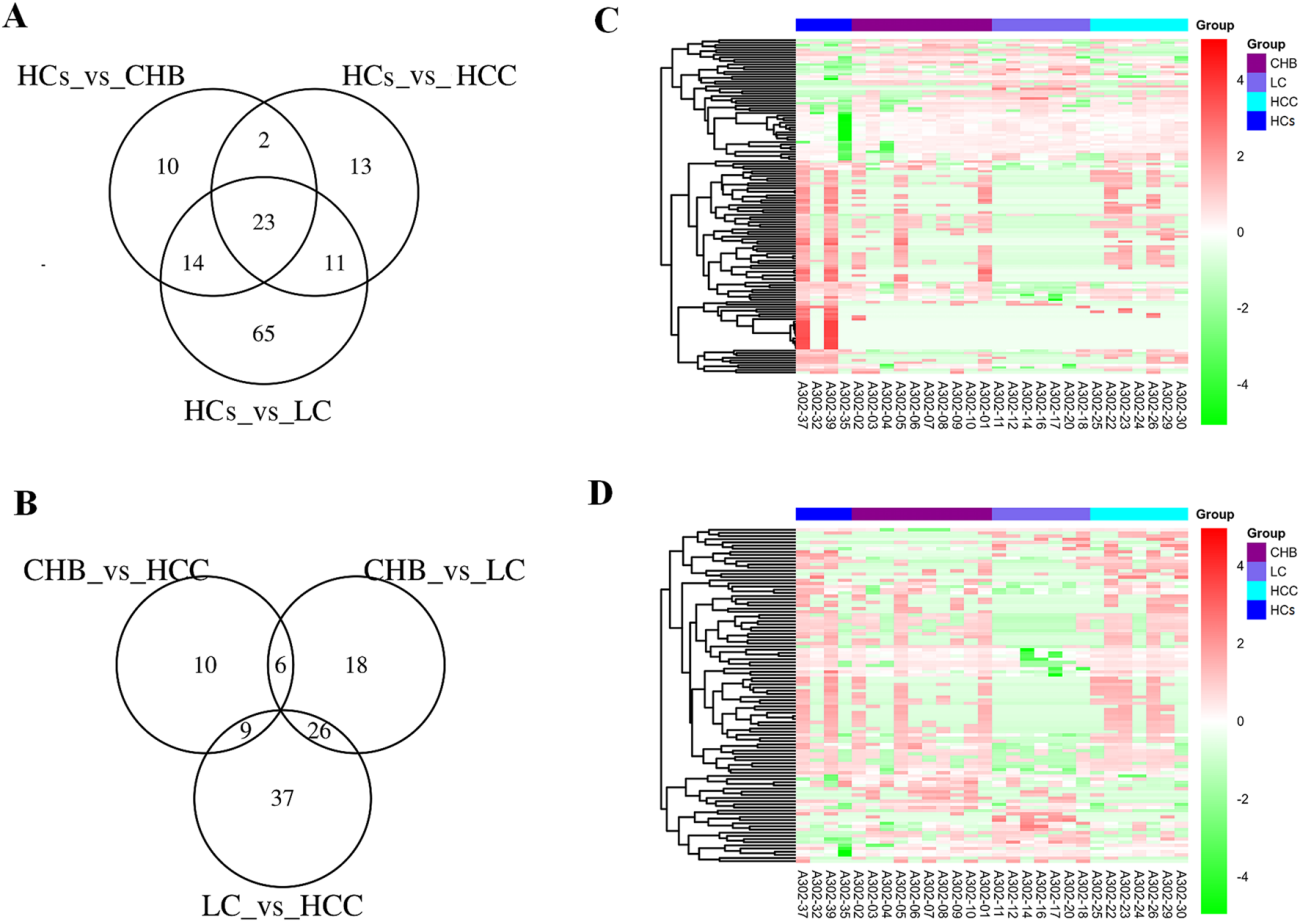
Protein profiles derived from exosome-enriched fractions of chronic hepatitis B (CHB), liver cirrhosis (LC), hepatocellular carcinoma (HCC), and healthy controls (HCs). (**A**) A Venn diagram showing pairwise comparison of differentially expressed proteins between patients with CHB, LC, and HCC against HCs. The Venn diagram was drawn by the R package VennDiagram. (**B**) A Venn diagram showing pairwise comparison of differentially expressed proteins among disease groups (CHB, LC, and HCC). The Venn diagram was drawn by the R package VennDiagram. (**C**) A clustergram of differentially expressed proteins analyzed through pairwise comparison between disease groups (CHB, LC, and HCC) and the HCs group. The clustergram was drawn by the R package Pheatmap. The ordinate represents proteins, and the abscissa represents samples. Clustering was performed using the values of log_10_ (label-free quantification + 1, LFQ + 1) and then clustering. On the left, lines in black show the hierarchical clustering of proteins. Colored bars at the top represent CHB, LC, HCC and HCs; the corresponding samples are shown at the bottom. Red and green represent proteins with high and low expression, respectively. (**D**) A clustergram of differentially expressed proteins for pairwise comparison between disease groups (CHB, LC, and HCC). The clustergram was drawn by the R package Pheatmap. The ordinate represents different proteins, and the abscissa represents different samples. Clustering was performed using the values of log_10_ (LFQ + 1). Red and green represent highly and lowly expressed proteins, respectively.

In addition, the HBV-related disease groups shared 23 identical differentially expressed proteins compared with HCs (Fig. 3a). The numbers of unique differentially expressed proteins for liver disease groups between CHB versus LC, LC versus HCC, and CHB versus HCC were 18, 37, and 10, respectively (Fig. 3b). Hierarchical cluster analysis was performed on the differentially expressed proteins, to cluster proteins with the same or similar expression. Cluster analysis can be used to group proteins according to their expression level, enabling more intuitive analysis of proteomic data and improving the accuracy of differentially expressed protein analysis. Cluster analysis revealed significant differences between the four groups of samples (Fig. 3c,d). All selected differentially expressed proteins were found to have statistically significant differences in expression (*P* ≤ 0.05).

### Comparison of differentially expressed proteins between the CHB, LC, HCC, and HCs groups

**Figure 4:**
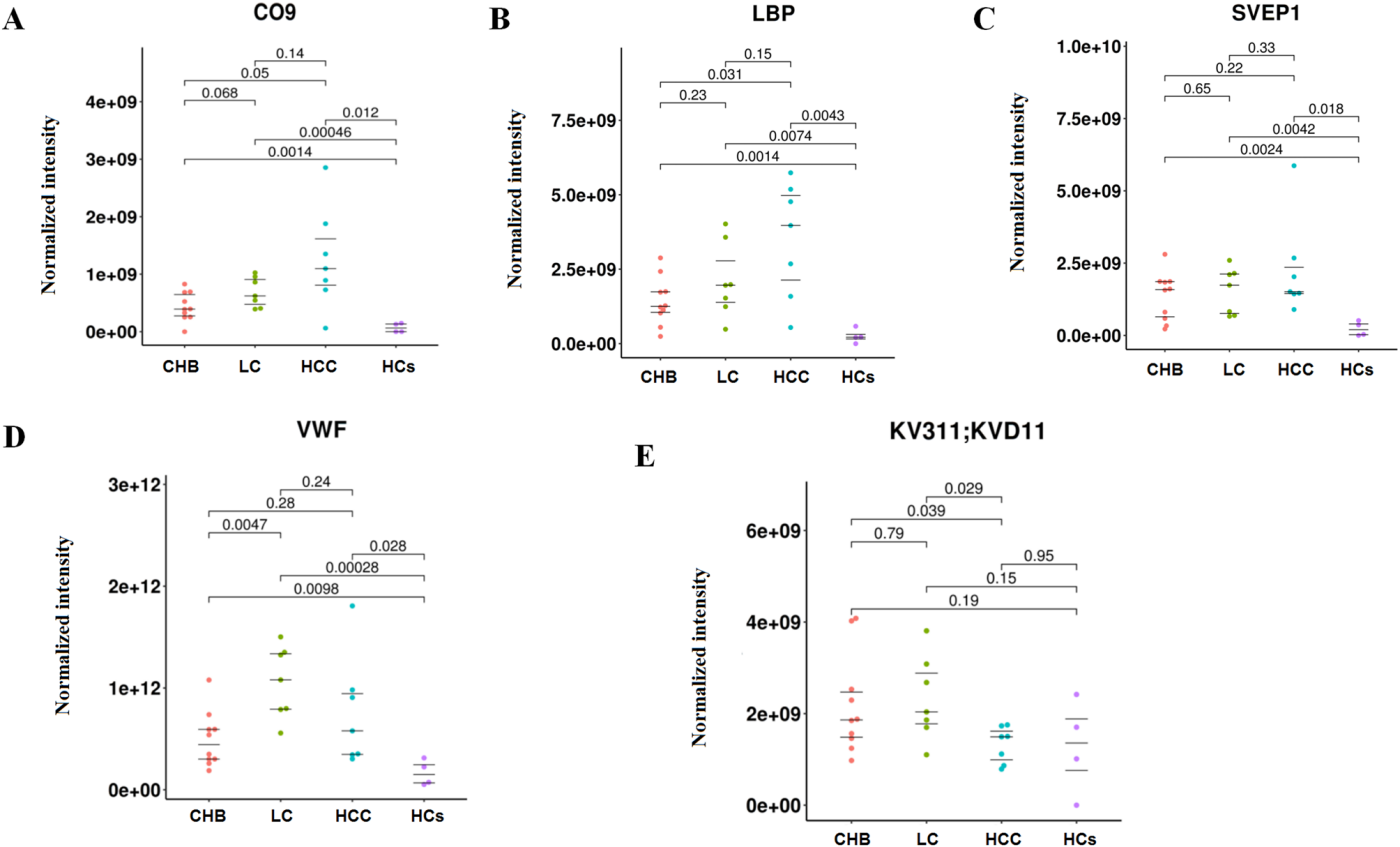
Comparison of the differentially expressed proteins, namely CO9, LBP, SVEP1, VWF and KV311, between the chronic hepatitis B (CHB), liver cirrhosis (LC), hepatocellular carcinoma (HCC), and healthy control (HC) groups (**A**–**E**). The main R package to draw the beeswarm plots was ggbeeswarm.

The expression levels of complement component C9 (CO9), lipopolysaccharide-binding protein (LBP); Sushi, von Willebrand factor type A, EGF, and pentraxin domain-containing protein 1 (SVEP1); and von Willebrand factor (VWF) in the EVs of patients with CHB, LC, and HCC were higher than those in HCs (*P* < 0.05). CO9 and LBP were significantly elevated in the HCC group, compared with the CHB group. And the expression levels of CO9 and LBP were gradually increasing from HCs, CHB, LC to HCC. In addition, The expression level of immunoglobulin kappa variable 3-11(KV311) was significantly low in the HCC group as compared with the CHB group or the LC group (*P* < 0.05). On the other hand, VWF was significantly elevated in the LC group as compared with the CHB group (*P* < 0.05), while SVEP1 was significantly elevated in the LC group as compared with the HCs group (*P* < 0.05) (Fig. 4a–e).

### Best candidate liquid biopsy biomarkers for diagnosing CHB, LC, and HCC

**Figure 5:**
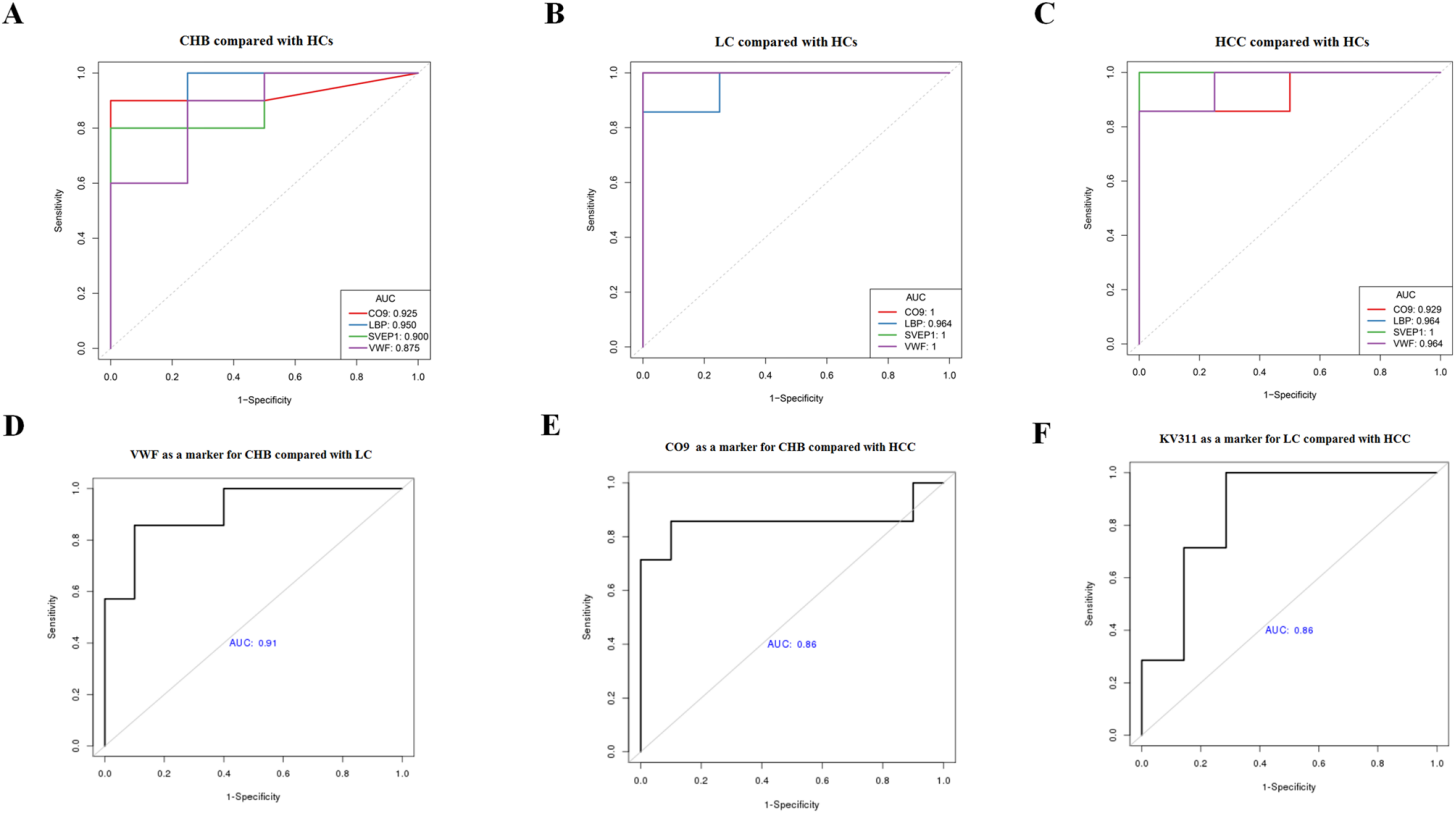
Diagnostic prediction (Receiver Operating Characteristics (ROC) curves and area under the curve (AUC) values) of the selected serum liquid biopsy biomarkers. AUC values were calculated by stepwise logistic regression algorithm, and then the graphs were drawn by R's own program. CO9, LBP, SVEP1 and VWF levels for diagnosing patients with chronic hepatitis B virus infection (CHB) (**A**), liver cirrhosis (LC) (**B**), and hepatocellular carcinoma (HCC) (**C**) compared with healthy controls (HCs). AUC values of VWF as a marker for diagnosing CHB compared with LC (**D**). AUC values of CO9 as a marker for diagnosing CHB compared with HCC (**E**). AUC values of KV311 as a marker for diagnosing LC compared with HCC (**F**).

Of the differentially expressed proteins, CO9, LBP, SVEP1, and VWF were the best candidate liquid biopsy biomarkers for diagnosing CHB, as their levels were increased in serum EVs (sEVs) and presented higher area under the curve (AUC) values of 0.925 (1.000/0.929), 0.950 (0.964/0.964), 0.900 (1.000/1.000), and 0.875 (1.000/0.964), respectively, in patients with LC and HCC compared with those of HCs (Fig. 5a–c). Of these, VWF had a better diagnostic efficiency for CHB and LC, with AUC values of 0.914 (Fig. 5d), whereas CO9 had a better diagnostic efficiency for CHB and HCC, with AUC values of 0.857 (Fig. 5e). KV311 was found to be a good biomarker, as its level also increased in sEVs and presented higher AUC values of 0.857 for the diagnosis of LC in comparison with those of HCC (Fig. 5f). Table 1 shows a panel of biomarkers that distinguish HCC from LC and CHB, as well as from healthy controls.

**Table 1:** The diagnostic capacity of the selected liquid biopsy biomarkers from serum extracellular vesicles (sEVs).

Characteristics	AUC	accuracy	SN	SP	PPV	NPV	*P* value
**CHB versus HCs**							
CO9	0.925	0.929	0.900	1.000	1.000	0.800	0.073
LBP	0.950	0.929	1.000	0.750	0.909	1.000	0.102
SVEP1	0.900	0.857	0.800	1.000	1.000	0.667	0.166
VWF	0.875	0.857	0.900	0.750	0.900	0.750	0.117
**LC versus HCs**							
CO9	1.000	1.000	1.000	1.000	1.000	1.000	1.000
LBP	0.964	0.909	1.000	0.750	0.875	1.000	0.295
SVEP1	1.000	1.000	1.000	1.000	1.000	1.000	0.999
VWF	1.000	1.000	1.000	1.000	1.000	1.000	0.999
**HCC versus HCs**							
CO9	0.929	0.909	0.857	1.000	1.000	0.800	0.270
LBP	0.964	0.909	1.000	0.750	0.875	1.000	0.401
SVEP1	1.000	1.000	1.000	1.000	1.000	1.000	0.999
VWF	0.964	0.909	1.000	0.750	0.875	1.000	0.359
**LC versus CHB**							
VWF	0.914	0.882	0.857	0.900	0.857	0.900	0.031
**HCC versus CHB**							
CO9	0.857	0.882	0.857	0.900	0.857	0.900	0.066
**HCC versus LC**							
KV311	0.857	0.857	1.000	0.714	0.778	1.000	0.099

*AUC* Area under the receiver operating characteristic curve, *CHB* Chronic hepatitis B, *HCs* Healthy controls, *HCC* Hepatocellular carcinoma, *LC* Liver cirrhosis, *SN* Sensitivity, *SP* Specificity, *PPV* Positive predictive value, *NPV* Negative predictive value.

### Target analysis of differentially expressed proteins in sEV-enriched fractions of CHB, LC, HCC, and HCs groups

**Figure 6:**
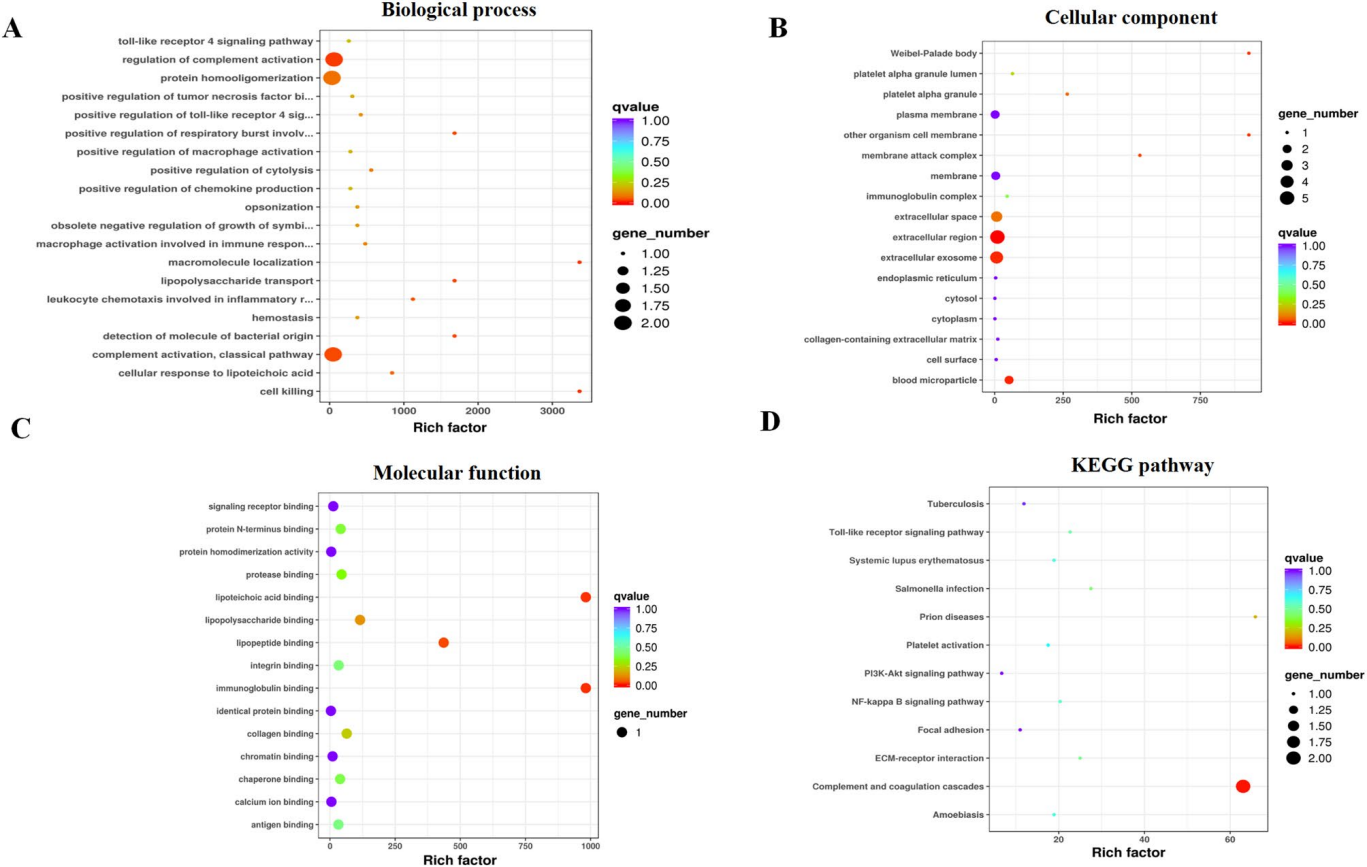
Gene Ontology (GO) and Kyoto Encyclopedia of Genes and Genomes (KEGG) enrichment analysis of differentially expressed proteins identified in serum EVs (sEVs). The Bubble plots were drawn by the R program. And the R program was written after enrichment significance, which was calculated by Fisher’s exact test. Bubble plots showing enrichment for the GO biological process (**A**), cellular component (**B**), and molecular function (**C**). A bubble plot showing enrichment for KEGG pathways (**D**).

We performed Gene Ontology (GO) enrichment of differentially expressed proteins identified in sEVs. Our findings revealed that the selected differentially expressed proteins were enriched in the biological process pathways of cell death, regulation of complement activation, and leukocyte chemotaxis in the inflammatory response; cellular components of the extracellular region and blood microparticle, and the membrane attack complex; and molecular function terms of lipoteichoic acid binding, immunoglobulin binding, and lipopeptide binding; and Kyoto Encyclopedia of Genes and Genomes (KEGG) pathways of complement and coagulation cascades (Fig. 6a–d)^16,17^.

## Discussion

Chronic HBV-related liver diseases, such as CHB, LC, and HCC, are highly prevalent in China; however, early and effective diagnostic markers that can be obtained non-invasively are lacking. Recently, exosomes have attracted increasing attention in chronic disease research^18,19^ and few studies have investigated the involvement of exosomes in the pathogenesis of the abovementioned diseases. In this study, we conducted a proteomic analysis of the isolated and purified serum exosomes from patients of CHB, LC and HCC, while using healthy individuals as controls. Our preliminary results provided a panel of differentially expressed proteins as promising biomarker candidates for the early and differential diagnosis of HBV-related liver diseases.

Considering the stated clinical difficulty in the differential diagnosis of CHB, LC, and HCC, we analyzed the differentially expressed protein profiles of plasma-derived exosomes of patients with the abovementioned diseases and HCs. The highest number of differentially expressed proteins was observed between LC and HCs, whereas the lowest was observed between CHB and HCC. Exosomes have a rich and complex protein composition, which may be involved in regulating a variety of biological processes. Therefore, exosomes may be considered as a new, specific, sensitive, and non-invasive diagnostic marker for HBV-related liver disease based on the differentially expressed proteins detected.

Label-free proteomics has substantially advanced the capacity for discovering new candidate biomarkers. We analyzed the proteomic profiles of patients with CHB, LC, and HCC and HCs using sEVs and identified five biomarkers, namely CO9, LBP, SVEP1, VWF, and KV311. CO9 plays a crucial role in the innate and adaptive immune response, as a component of the membrane attack complex which forms pores in the plasma membrane of target cells^20^. LBP, a glycolipid found in the outer membrane of all gram-negative bacteria, binds to a portion of bacterial lipopolysaccharide. LBP plays a significant role in the innate immune response^21^. SVEP1 plays a crucial role in regulating intercellular adhesion^22^. VWF, a multimeric glycoprotein synthesized primarily by endothelial cells^23^, is involved in platelet adhesion and aggregation and other hemostatic mechanisms^24^. KV311 is involved in antigen recognition via the V region of immunoglobulin light chain variable domains^25^.

Our results indicated that the expression of CO9, LBP, SVEP1, and VWF in the sEVs of CHB, LC, and HCC was significantly higher than that in HCs. Furthermore, these markers were associated with excellent diagnostic accuracy for CHB, LC, and HCC. The AUC values of CO9, LBP, SVEP1, and VWF for HCC were 0.929, 0.964, 1.000, and 0.964, respectively, whereas the AUC values of AFP for HCC were reported to be 0.830 in a systematic review and meta-analysis^26^. Obviously, the AUC values of these novel biomarker candidates were significantly higher than AFP’s for HCC. Moreover, LBP expression in the sEVs from the HCC group was higher than that in the CHB group. These findings are consistent with those of the study by Cai et al., who reported that serum AFP combined with LBP, which is highly expressed in HCC, may predict poor prognosis after curative resection for HCC^27^. Chen et al. showed that in the virus-associated tumor microenvironment, LBP may significantly realize anti-tumor effects through the NF-κB-associated signaling pathway^28^. Li et al. showed that LBP promotes the anti-proliferative and apoptotic effects of 125I, which provides a solid foundation for better combination therapy^29^. Furthermore, LBP could maintain high levels of T cells in tumor tissue, tumor-draining lymph nodes, and peripheral blood. LBP can inhibit the increase of Treg populations and the production of interleukin-10 (IL-10) and transforming growth factor-beta 1 (TGF-β1) in the serum, mitigate the exhaustion of T cells, promote the infiltration of CD8^+^ T cells in tumor tissue, and maintain the cytotoxicity of lymphocytes^30^. LBP may, therefore, play an important role in the diagnosis and treatment of HCC.

KV311 and CO9 may be used as complements of LBP for the diagnosis and evaluation of HCC. We found that KV311 expression in the sEVs of the HCC group was significantly lower than that in the CHB and LC groups. Thus, KV311 seems to have a better diagnostic efficiency for HCC than LC, with AUC values of 0.857, whereas CO9 seems to have a better diagnostic efficiency for HCC than CHB, with AUC values of 0.857. KV311, which has rarely been reported, is mainly involved in humoral immunity, with the V region of the variable domain of immunoglobulin light chains^25^. Studies have shown that B cells can recognize antigens and regulate antigen processing and presentation. B cells also regulate T-cells and innate immune responses^31^, and represent a heterogeneous population with functionally distinct subsets, which play a role in protumor and antitumor immune responses. The balance between B cell subtypes may influence tumor development and behavior^32,33^. Zhang et al. found that B cells may produce cell surface molecules that block the targeting and destruction of tumor cells, or produce inhibitors that inhibit the response of other immune cells^34^. CO9 is a member of the complement system, which provides effective immune surveillance and homeostasis. CO9 is important for innate and adaptive immune responses^35^. Using label-free proteomics and CO9 immunoblotting, Chong et al. reported that CO9 levels were significantly higher in patients with gastric cancer than HCs^36^. Zhang et al. also reported that complement activation promotes tumorigenesis and tumor progression in the tumor microenvironment, and the expression of complement proteins increased in malignant tumors^37^. However, there have been no reports on HCC with CO9. Our findings revealed that LBP, KV311, and CO9 may be used in combination as new indicators for the diagnosis and treatment evaluation of HCC. SVEP1, a cell adhesion molecule, plays a central role in the regulation of intercellular adhesion^22^. Chen et al. showed that SVEP1 was involved in mediating the proliferation and metastasis of HCC by downregulating SVEP1 expression and activating the PI3k/Akt signaling pathway through miR-1269b^38^. Our research shows that SVEP1 may be not a promising biomarker for the diagnosis of HBV-related liver diseases at different stages, because its expression is just higher than the HCs group. SVEP1 expression may be one of universal markers for HCC and liver fibrosis, but it is not classify HBV-related liver diseases at different stages.

Our findings revealed that VWF may be a predictor of cirrhosis. VWF expression in the sEVs of patients with LC was higher than that in patients with CHB. VWF seems to have a better diagnostic efficiency for LC than CHB, with an AUC value of 0.914. GO enrichment of differentially expressed proteins revealed that the main function of VWF is immunoglobulin binding, and KEGG enrichment analysis of differentially expressed proteins showed that VWF is mainly involved in complement and coagulation cascades. Wu et al. found that an increase in VWF level in liver tissues may cause an increase in VWF level in the plasma. In addition, as a noninvasive biomarker, VWF can predict portal hypertension and esophageal varices in patients with HBV infection and cirrhosis^39^. VWF was reported to be associated with the progression of LC in chronic hepatitis and was negatively correlated with platelet count, prothrombin time, and albumin level^40^. Moreover, LC was reported to be involved in angiogenesis^41^, whereas VWF suppresses angiogenesis^42–44^. VWF may therefore be related to the progression of liver fibrosis.

To the best of our knowledge, this is the first report profiling differentially expressed proteins in plasma-derived exosomes of patients with CHB, LC, and HCC in comparison to HCs. Moreover, we identified novel, non-invasive, and specific biomarkers for diagnosing the progression of CHB. Our results demonstrate that LBP, KV311, and CO9 could be used in combination as indicators for the diagnosis and treatment evaluation of HCC. SVEP1 and VWF may be promising biomarkers for auxiliary diagnosis of HCC and liver fibrosis. Our preliminary results provide insights into the value of exosomes as candidate biomarkers for predicting the progression of HBV-related liver diseases.

## Methods

### Blood sample collection

Twenty-four patients with chronic HBV infection were enrolled at the First Affiliated Hospital of Medical College, Zhejiang University, Hangzhou, China between October 2020 and November 2020. The patients were divided into 3 groups, including 10 with CHB, 7 with LC, and 7 with HCC, according to the practice guidelines established by the American Association for the Study of Liver Diseases^6^ and the guideline established by the Chinese Medical Association (Chinese Society of Hepatology and Chinese Society of Infectious Diseases)^45^. An additional four HCs were included in the study. The information of enrolled cases is listed in Table 2. Exclusion criteria included patients with other chronic liver diseases related to drugs, alcohol, or autoimmune diseases. Patients infected with hepatitis A, C, or D virus, or human immunodeficiency virus, were excluded. This study was approved by the Ethics Review Committee of the First Affiliated Hospital, School of Medicine, Zhejiang University. All volunteers provided written informed consent and a questionnaire for participation in the study, in accordance with the Declaration of Helsinki.

**Table 2:** Clinical characteristic of the subjects.

	HCs (n = 4)	CHB^#^ (n = 10)	LC (n = 7)	HCC^##^ (n = 7)
Age (years)	31.50 ± 6.98	46.30 ± 14.59	52.71 ± 10.11	52.86 ± 7.26
Sex (male/female)	2/2	8/2	6/1	6/1
HBV DNA (log_10_ copies/mL)	–	6.20 ± 1.98	4.95 ± 2.21	4.98 ± 2.11
ALT (U/L)	32.25 ± 28.42	367.40 ± 313.05	55.00 ± 25.47	60.86 ± 60.18
AST (U/L)	24.00 ± 17.42	216.40 ± 226.92	105.57 ± 53.53	76.14 ± 52.37
GGT (U/L)	26.25 ± 12.93	135.60 ± 70.66	356.14 ± 579.36	205.86 ± 87.05

Data are expressed as the means ± standard deviation or n.

*ALT* Alanine aminotransferase, *AST* Aspartate aminotransferase, *CHB* Chronic hepatitis B, *HCs* Healthy controls, *HCC* Hepatocellular carcinoma, *LC* Liver cirrhosis, *GGT* Gamma glutamyl transferase.

^#^Treatment-naïve active CHB patients.

^##^Newly diagnosed cases of HCC without any treatment.

### Exosome isolation

The exosomes were isolated through size exclusion chromatography^46^. Briefly, 0.8 μm-filtered blood plasma (1 mL) was 1.5-fold diluted with phosphate-buffered saline (PBS) and purified using Exosupur columns (Echo Biotech, China). Then, the samples were eluted with PBS (0.1 M). Eluate fractions (2 mL) were collected, according to the manufacturer’s instructions, and concentrated to 200 μL through 100 kDa molecular weight cut-off Amicon®Ultra spin filters (Merck, Germany).

#### NTA

Vesicle suspensions with concentrations (between 1 × 10^7^ and 1 × 10^9^/mL) were examined using the ZetaView PMX 110 (Particle Metrix, Meerbusch, Germany), which was equipped with a 405 nm laser, to determine the quantity and size of particles isolated. A video of 60 s duration was taken with a frame rate of 30 frames/s, and article movement was analyzed using the NTA software (ZetaView 8.02.28).

#### TEM

For the characterization of exosomes, isolated fractions of the exosomes were analyzed using TEM. Briefly, exosome fraction (10 μL) was placed on a copper mesh and incubated at 37 °C for 1 min. The exosomes were contrasted using uranyl acetate solution for 1 min, washed with sterile distilled water, and dried for 2 min under incandescent light. Finally, the copper mesh was observed and photographed using a transmission electron microscope (H-7650, Hitachi Ltd., Tokyo, Japan).

### WB analysis

The concentration of exosomal proteins was detected using the bicinchoninic acid (BCA) Protein Assay Kit. Based on the quantitative results, the exosome supernatant was denatured in 5 × sodium dodecyl sulfonate (SDS) buffer in proportion and subjected to WB analysis (10% SDS–polyacrylamide gel electrophoresis; 50 µg protein/lane) using rabbit polyclonal antibody CD9 (60232-1-Ig, Proteintech, Rosemont, IL, USA), TSG101 (diluted 1:500; cat. no. abs115706; Absin Bioscience Inc., Shanghai, China), CD63 (sc-5275, Santa Cruz, CA, USA), and calnexin (10427-2-AP, Proteintech, Rosemont, IL, USA).

### Label free-methods

#### Total protein extraction

Isolated exosomes were suspended in lysis buffer containing urea (6 M), NH_4_HCO_3_ (100 mM), and 0.2% SDS; ultrasonicated on ice; and centrifuged at 12,000 × *g* for 15 min at 4 °C. The supernatant was reduced with 10 mM dithiothreitol (DTT) for 1 h at 56 °C and alkylated with iodoacetamide for 1 h at 25 °C in the dark. Then, the samples were mixed with 4 volumes of acetone and incubated at − 20 °C for 2 h. The precipitate was collected after centrifugation (12,000 × g for 15 min)and washed with cold acetone. The pellet was dissolved in triethylammonium bicarbonate (TEAB) (0.1 M) buffer and urea (6 M).

### Protein quality test

The protein quality of the samples was evaluated through a bovine serum albumin (BSA) standard protein curve using the Pierce BCA Protein Assay Kit (Product No. 23225, Thermo Scientific, USA). To the standard sample (10 μL), BCA solution (200 μL) was added to each well of a 96-well plate. The plate was covered and incubated at 37 °C for 30 min. The standard curve was used to measure the protein concentration of each isolated sample at 562 nm using a plate reader. Each sample solution was prepared at a different dilution in triplicate. Then, the protein sample (20 μL) was loaded onto a 12% SDS-PAGE gel for electrophoresis. The separating gel was run at 120 V for 90 min and the stacking gel at 80 V for 20 min. The gel was stained with Coomassie brilliant blue R-250 and destained until the bands were visualized clearly.

### Trypsin treatment

We added trypsin (3 μL, 1 μg/μL) and TEAB buffer (500 μL, 100 mM) to each protein sample, and incubated the mixture at 37 °C overnight. The digested sample was mixed with an equal volume of 1% formic acid and centrifuged at 12,000 × *g* for 5 min at room temperature. The supernatant was slowly loaded onto the C18 desalting column, washed three times with wash solution (1 mL, 0.1% formic acid and 4% acetonitrile), and eluted two times with elution buffer (0.4 mL, 0.1% formic acid and 75% acetonitrile). The eluates were pooled and lyophilized.

### Liquid chromatography-tandem mass spectrometry (LC–MS/MS) analysis

The lyophilized protein powder was dissolved in 0.1% formic acid (10 μL) in water (solvent A), and then injected into a home-made C18 Nano-Trap column (2 cm × 75 μm, 3 μm). Then, using a mobile phase of 0.1% formic in 80% acetonitrile (solvent B), peptides were separated in a homemade analytical column (15 cm × 150 μm, 1.9 μm). Sample elution was performed at a flow rate of 600 nL/min by increasing the concentration of solvent B from 6 to 100% in 60 min. In addition, the separated peptides were analyzed using the Q Exactive HF-X mass spectrometer (Thermo Fisher), with the Nanospray Flex (ESI) ion source and a spray voltage of 2.3 kV.

MS raw data were retrieved from the UniProt database (http://www.uniprot.org); oxidation of methionine and acetylation of the N-terminus were specified as variable modifications, whereas carbamidomethylation was specified as a fixed modification. The identified protein contained at least 1 unique peptide with a false discovery rate of no more than 0.01. Then, GO analysis was performed to evaluate the potential roles of differentially expressed proteins in biological processes, as cellular components, and in molecular functions. The databases of Clusters of Orthologous Groups and KEGG were used to annotate protein families and pathways.

### Statistical analysis

For the detection of differentially expressed proteins, we used the limma package in R^47^ to analyze differences between groups of duplicate samples. MaxQuant software was used for protein identification and relative quantification. Swissprot Database was used to annotate proteins. Blast software was used to compare protein sequences with NR (Non-Redundant Protein Sequence) database. According to the corresponding relationship between NR and GO provided by the UCSC database, the results of protein GO annotation were obtained. The enrichment factor was calculated as follows:

$$ {\text{Enrichment}}\;{\text{factor}} = \frac{{{\text{Number}}\;{\text{of}}\;{\text{DEPs}}\;{\text{in}}\;{\text{the}}\;{\text{pathway}}/{\text{Number}}\;{\text{of}}\;{\text{all}}\;{\text{the}}\;{\text{proteins}}\;{\text{in}}\;{\text{the}}\;{\text{pathway}}}}{{{\text{Number}}\;{\text{of}}\;{\text{DEPs}}\;{\text{which}}\;{\text{were}}\;{\text{annotated}}\;{\text{to}}\;{\text{KEGG}}/{\text{Number}}\;{\text{of}}\;{\text{proteins}}\;{\text{which}}\;{\text{were}}\;{\text{annotated}}\;{\text{to}}\;{\text{KEGG}}}} $$

Differences were considered significant when *P* ≤ 0.05 and fold change > 1.1. Analysis of differences between groups was performed using *t*-tests.

## Supplementary Information

Supplementary Information 1.

Supplementary Information 2.

Supplementary Information 3.

Supplementary Information 4.

Supplementary Information 5.

## Data Availability

The datasets used and/or analyzed during the current study are available from the corresponding author on reasonable request.
