# Supplementary material for: Differential proteomic analysis of plasma-derived exosomes as diagnostic biomarkers for chronic HBV-related liver disease
Source: Sci Rep. 2022 Aug 24;12:14428. doi: 10.1038/s41598-022-13272-4 (PMC9402575; doi:10.1038/s41598-022-13272-4)
Supplement: Supplementary file 1 — Supplementary Information 1. [file 41598_2022_13272_MOESM1_ESM.doc]

The original gel graph contains three samples, only the the middle one (which highlight in red rectangle) is used in this paper. The other two samples are involved in other irrelevant experiments.
