# Supplementary figures and images for: Differential proteomic analysis of plasma-derived exosomes as diagnostic biomarkers for chronic HBV-related liver disease
Source: Sci Rep. 2022 Aug 24;12:14428. doi: 10.1038/s41598-022-13272-4 (PMC9402575; doi:10.1038/s41598-022-13272-4)

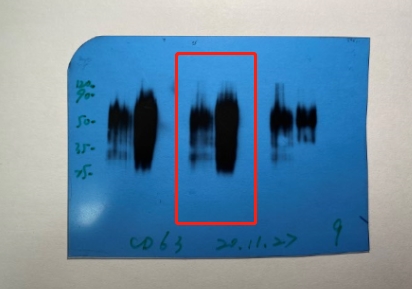

Supplement: Supplementary file 2 — Supplementary Information 2. [file 41598_2022_13272_MOESM2_ESM.jpg]

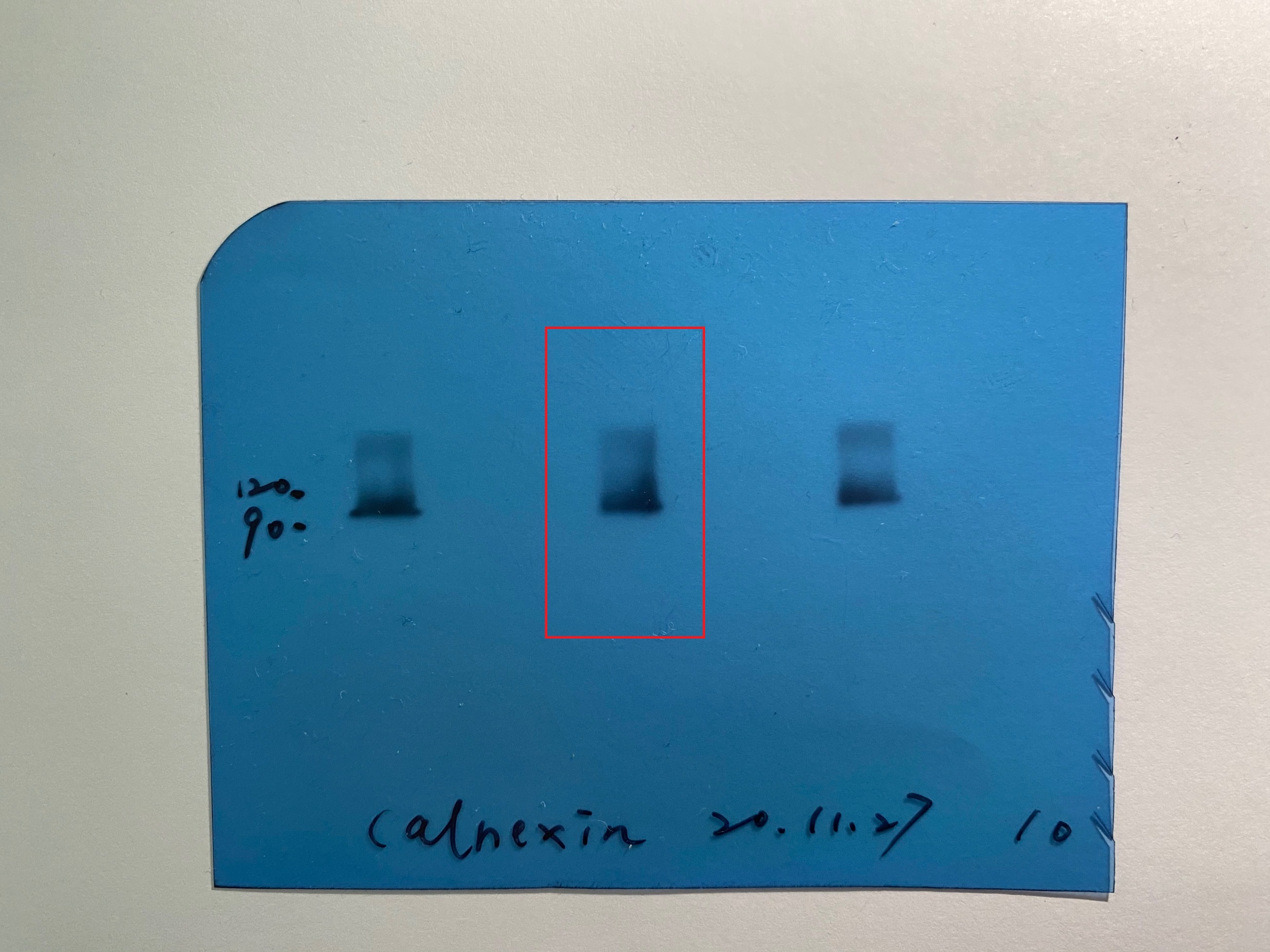

Supplement: Supplementary file 3 — Supplementary Information 3. [file 41598_2022_13272_MOESM3_ESM.jpg]

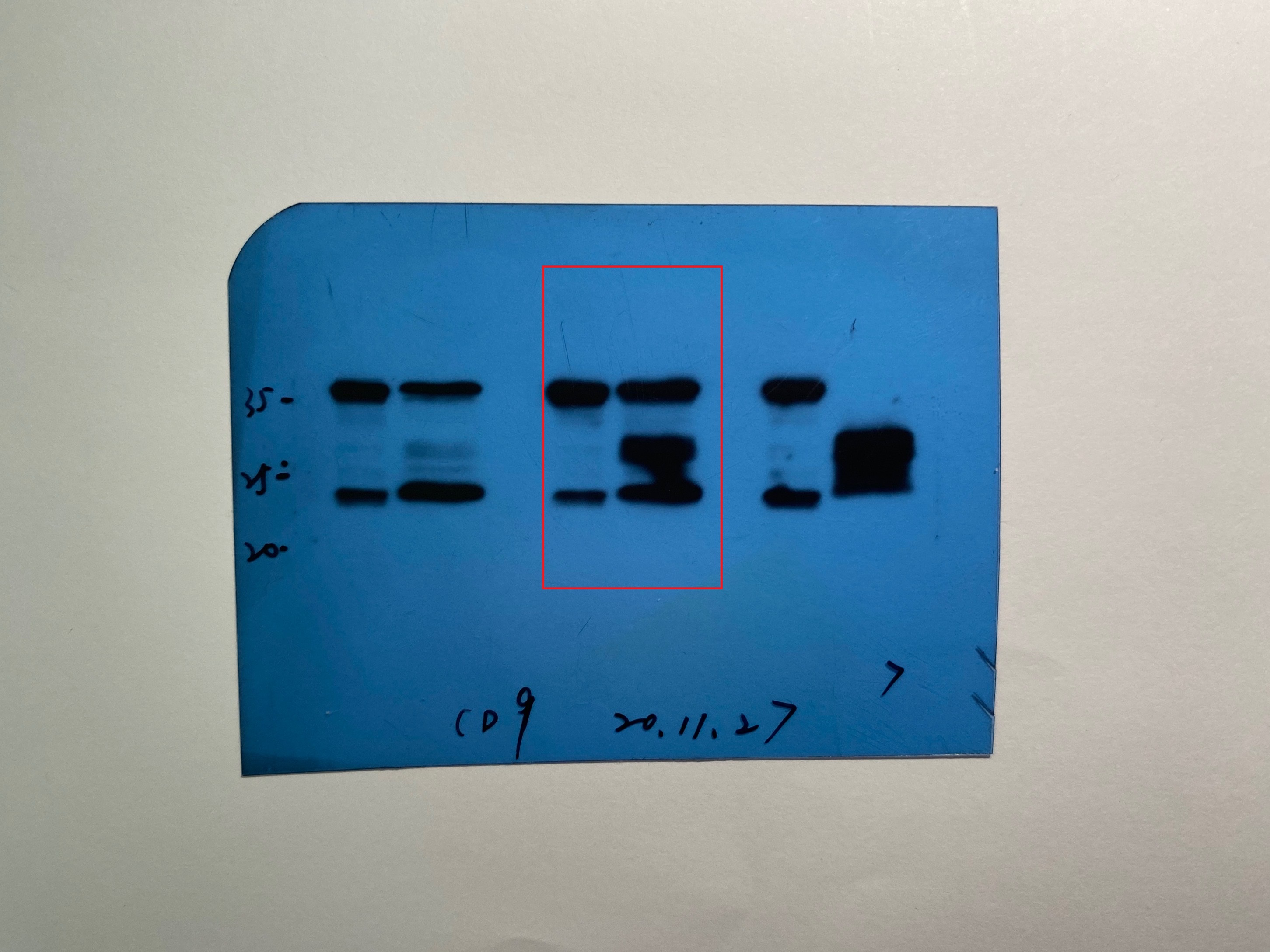

Supplement: Supplementary file 4 — Supplementary Information 4. [file 41598_2022_13272_MOESM4_ESM.jpg]

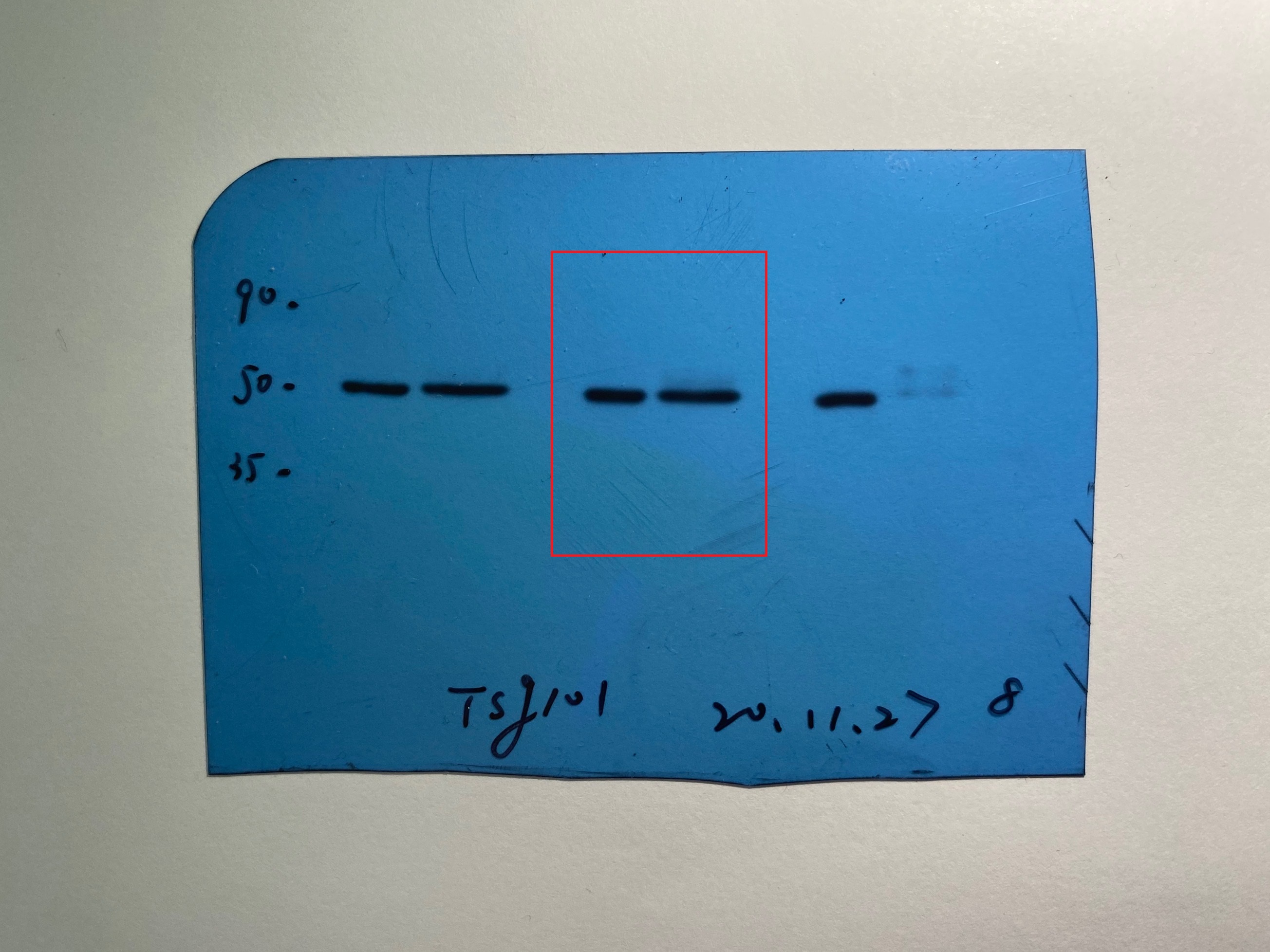

Supplement: Supplementary file 5 — Supplementary Information 5. [file 41598_2022_13272_MOESM5_ESM.jpg]
